# The Diagnostic Value of D-Dimer Testing in the Differential Diagnosis of Acute Abdomen in the Emergency Department

**DOI:** 10.3390/jcm15145660

**Published:** 2026-07-19

**Authors:** Dimitrios A. Chatzelas, Georgios V. Tsamourlidis, Apostolos G. Pitoulias, Georgios A. Pitoulias

**Affiliations:** 1 Division of Vascular Surgery, 2nd Department of Surgery, Faculty of Medicine, “G. Gennimatas” General Hospital of Thessaloniki, School of Health Sciences, Aristotle University of Thessaloniki, 54124 Thessaloniki, Greece; tsamougv@gmail.com (G.V.T.); pitoulias@yahoo.com (G.A.P.); 2 Department of Vascular and Endovascular Surgery, St Bernward Hospital, 31134 Hildesheim, Germany; appitoulias@yahoo.com

**Keywords:** acute abdomen, emergency department, D-dimer, diagnostic biomarker, differential diagnosis

## Abstract

Acute abdominal pain is one of the most common presentations in the emergency department, and encompasses a broad spectrum of conditions, ranging from self-limiting inflammatory diseases to life-threatening vascular emergencies. Although D-dimer is well established in the diagnostic work-up of venous thromboembolism and acute aortic syndromes, its role in the evaluation of acute abdomen remains incompletely defined. This narrative review summarizes and critically appraises the current evidence regarding the diagnostic utility of D-dimer across the major causes of acute abdomen. Available data indicate that D-dimer has its greatest clinical value in vascular and ischemic conditions, particularly acute mesenteric ischemia, where its high sensitivity may support rule-out strategies in carefully selected low-risk patients. In contrast, although D-dimer concentrations are frequently elevated in appendicitis, pancreatitis, bowel obstruction, diverticulitis, and gynecological emergencies, its limited specificity precludes its use as a primary diagnostic test in these disorders. Based on the available evidence, we propose a practical emergency department diagnostic framework emphasizing selective rather than routine D-dimer testing. D-dimer should be regarded as an adjunct to clinical evaluation and appropriate imaging, not a stand-alone diagnostic test, with its greatest contribution being the early recognition or exclusion of time-critical vascular abdominal emergencies.

## 1. Introduction

Acute abdominal pain is among the most frequent and diagnostically complex presentations in Emergency Medicine. The term “acute abdomen” is clinically useful, but pathophysiologically broad, encompassing inflammatory disorders, such as acute appendicitis, cholecystitis, diverticulitis, and pancreatitis; obstructive disorders, such as small bowel obstruction and incarcerated hernia; perforated hollow viscus; ischemic disorders, such as acute mesenteric ischemia (AMI), and strangulated obstruction; vascular emergencies, such as ruptured abdominal aortic aneurysm (RAAA), and aortic dissection (AD); urologic conditions, such as renal colic, and pyelonephritis; gynecologic emergencies, such as ectopic pregnancy, and adnexal torsion; and extra-abdominal mimics, such as pulmonary embolism (PE) and myocardial infarction (MI) [1,2]. Because the early symptoms of several diseases may be non-specific, emergency physicians often combine clinical examination, laboratory tests, ultrasonography, computed tomography (CT), computed tomography angiography (CTA), and surgical or vascular consultation [1,3].

D-dimer is produced when plasmin degrades cross-linked fibrin, and therefore reflects recent or ongoing activation of coagulation and fibrinolysis [4]. Its best-established use in Emergency Medicine is the exclusion of venous thromboembolism (VTE) in patients with low or intermediate pre-test probability [4,5]. This diagnostic logic has prompted interest in D-dimer for acute abdomen, particularly because several abdominal emergencies involve thrombosis, ischemia, bowel necrosis, endothelial injury, or systemic inflammatory coagulation activation [6,7,8]. However, D-dimer is not disease-specific. It may be elevated in older age, infection, malignancy, pregnancy, trauma, postoperative states, renal dysfunction, disseminated intravascular coagulation, inflammatory disease, and critical care [4,5]. Consequently, the central question is not whether D-dimer is abnormal in acute abdomen, but whether it can improve differential diagnosis, risk stratification, or guide decision about urgent imaging.

The aim of this narrative review is to synthesize the available evidence on the diagnostic value of D-dimer testing in patients presenting to the emergency department with acute abdomen. The review focuses on disease-specific interpretation, reported diagnostic thresholds and performance, where available, and the practical role of D-dimer in differentiating the various causes of acute abdominal pain. Particular emphasis is taken on how D-dimer should be integrated with pre-test probability, clinical red flags, other laboratory markers, and imaging, rather than used as an isolated diagnostic test.

## 2. Biological Rationale for D-Dimer Elevation in Acute Abdomen

D-dimer elevation in acute abdomen may occur through several mechanisms. Primary intravascular thrombosis directly generates fibrin turnover in mesenteric arterial thrombosis, mesenteric venous thrombosis (MVT), portal vein thrombosis (PVT), renal infarction, splenic infarction, PE, and AD [4,6,7]. Bowel ischemia and necrosis can trigger endothelial injury, tissue factor release, microvascular thrombosis, mucosal barrier disruption, and systemic inflammatory activation, all of which can increase fibrin formation and fibrinolysis [6,8,9]. Severe inflammatory diseases, such as pancreatitis, complicated appendicitis, peritonitis, and sepsis may also elevate D-dimer without a primary macroscopic thrombus [10,11,12]. This explains why D-dimer can be abnormal in many serious abdominal diseases, but cannot usually identify the specific diagnosis.

## 3. D-Dimer in Undifferentiated Non-Traumatic Acute Abdomen

Several studies have evaluated D-dimer as a broad marker of surgical pathology in patients presenting with non-traumatic acute abdomen. Akyildiz et al. performed a prospective study of 225 patients with non-traumatic acute abdominal pain, and compared D-dimer with leukocyte count for distinguishing patients requiring immediate laparotomy from those managed non-operatively [13]. The authors reported that D-dimer had higher sensitivity than leukocyte count for immediate laparotomy, with a sensitivity of 95.7%, compared to 74.8% for leukocyte count [13]. In the same study, a D-dimer level around three times the normal value predicted surgical pathology with 64% sensitivity and 86% specificity, while patients requiring immediate laparotomy had a mean D-dimer value of 4.49 ± 3.00 μg/mL [13].

These findings are clinically relevant, because they suggest that D-dimer may behave as a global marker of serious intra-abdominal pathology, particularly when ischemia, necrosis, or inflammatory coagulation activation is present [13]. However, this does not mean that D-dimer is a general diagnostic test for acute abdomen. The outcome “need for immediate laparotomy” depends on local surgical thresholds, imaging access, and clinical practice, and the test does not identify whether the underlying disease is appendicitis, bowel obstruction, ischemia, perforation, or another condition [13]. Later work evaluating combinations of leukocyte count and D-dimer suggested that combined models may improve discrimination compared with either variable alone, but such models are not widely validated, and are not incorporated into major acute abdomen algorithms [14].

Assay interpretation is also important. D-dimer concentrations are reported using either fibrinogen equivalent units (FEU) or D-dimer units (DDU), which are not interchangeable. Because one molecule of cross-linked fibrinogen generates approximately one-half its mass as D-dimer fragments, DDU values are approximately half the corresponding FEU values. Consequently, the commonly used diagnostic threshold of 500 ng/mL FEU is equivalent to approximately 250 ng/mL DDU [4,5]. Unfortunately, many publications report numerical cut-offs without specifying the reporting units, making direct comparison between studies difficult and potentially leading to misinterpretation in clinical practice. Unless otherwise specified, the thresholds reported in this review are presented exactly as described in the original publications. Furthermore, age-adjusted thresholds, commonly calculated as age × 10 ng/mL FEU in patients older than 50 years, have been validated for venous thromboembolism but not for acute abdominal emergencies. Therefore, D-dimer values should always be interpreted according to the assay methodology and reporting units used by the individual laboratory [4,5,9].

## 4. Acute Mesenteric Ischemia

AMI is the abdominal emergency for which D-dimer has the strongest disease-specific evidence. It is uncommon, but highly lethal, and delayed diagnosis remains a major cause of mortality [3,6]. The rationale for D-dimer testing in AMI is strong, because arterial embolism, arterial thrombosis, MVT, bowel wall ischemia, and systemic inflammatory activation can all increase fibrin turnover [6,8,15]. Acosta et al. reported in an early clinical study that D-dimer was frequently elevated in patients with acute bowel ischemia, and suggested that it might be useful as an early marker, although the study was preliminary and required validation [16]. A systematic review found that D-dimer had a pooled sensitivity of 96% and specificity of 40% for AMI, indicating potential value for exclusion, but poor value for confirmation [6]. Sun et al. later performed a meta-analysis focused on AMI and reported a pooled sensitivity of 0.94 and specificity of 0.50 [8]. These estimates are consistent: D-dimer is usually sensitive, but its specificity is too low for rule-in diagnosis.

More recent systematic reviews have been more cautious. Reintam Blaser et al. evaluated biomarkers for AMI and concluded that no biomarker, including D-dimer, had adequate accuracy to replace imaging [15]. In that review, D-dimer showed only moderate predictive value for transmural ischemia, and the authors emphasized high risk of bias, selected study populations, heterogeneous timing of measurement, and insufficient standardization [15]. Blauw et al. similarly concluded that the diagnostic value of biomarkers in AMI remains insufficiently substantiated [17]. The World Society of Emergency Surgery guidelines emphasize that no laboratory test can reliably exclude AMI when clinical suspicion is high, and that CTA should be performed without delay in suspected cases [3]. The European Society for Vascular Surgery guidelines similarly highlight the need for urgent imaging and revascularization when appropriate [18].

For emergency practice, D-dimer should be interpreted according to pre-test probability. A normal D-dimer may reduce the likelihood of AMI in a low-risk patient with non-specific abdominal pain, stable physiology, no atrial fibrillation (AF), no severe atherosclerotic disease, no metabolic acidosis, no peritonitis, and no alarming CT findings [6,8]. However, D-dimer must not be used to delay CTA in patients with pain out of proportion to examination, AF, known peripheral or coronary atherosclerosis, recent MI, heart failure, hypotension, gastrointestinal bleeding, elevated lactate, metabolic acidosis, peritonitis, or shock [3,18].

## 5. Mesenteric Venous Thrombosis and Portal Vein Thrombosis

MVT and PVT are important causes of acute abdomen and may present with abdominal pain that is initially disproportionate to physical signs, similar to arterial mesenteric ischemia [18]. D-dimer is biologically plausible in this setting, because venous thrombosis directly generates cross-linked fibrin degradation products [4,18]. However, the evidence base is less robust than for VTE of the legs and lungs, and D-dimer cannot localize the thrombus or distinguish MVT/PVT from other thrombotic or inflammatory disorders.

In practice, elevated D-dimer may support suspicion of MVT or PVT in a patient with abdominal pain and risk factors, such as thrombophilia, malignancy, cirrhosis, pancreatitis, intra-abdominal infection, inflammatory bowel disease, oral contraceptive use, pregnancy, or recent surgery [18]. Nevertheless, diagnosis requires contrast-enhanced CT, and treatment decisions depend on the extent of thrombosis, bowel viability, bleeding risk, and underlying cause [18]. A normal D-dimer, on the other hand, should be interpreted cautiously, particularly when symptoms are prolonged or clinical suspicion remains substantial.

## 6. Acute Aortic Syndromes Presenting as Acute Abdomen

AAS include AD, intramural hematoma, penetrating aortic ulcer, and rupture or impending aortic rupture [7,19]. These conditions may present with chest pain, back pain, abdominal pain, flank pain, syncope, lower limb ischemia, visceral malperfusion, neurologic symptoms, or shock [19]. Because abdominal and flank presentations can mimic renal colic, pancreatitis, biliary disease, AMI, or non-specific abdominal pain, AAS is an essential vascular differential diagnosis in acute abdomen.

D-dimer has been studied extensively in acute AD. Watanabe et al. performed a systematic review and meta-analysis of D-dimer for AAS, and reported high diagnostic sensitivity, although specificity was limited [7]. The ADvISED prospective multicenter study evaluated a diagnostic strategy combining the Aortic Dissection Detection Risk Score (ADD-RS) with D-dimer [20]. In that study, D-dimer was considered negative below 500 ng/mL FEU, and the combination of low clinical probability and negative D-dimer had very high negative predictive value [20]. Specifically, strategies using ADD-RS = 0 plus D-dimer < 500 ng/mL FEU or ADD-RS ≤ 1 plus D-dimer < 500 ng/mL FEU were proposed as possible rule-out strategies in selected patients [20]. External reviews have similarly supported combined clinical probability and D-dimer strategies, while warning against D-dimer as a stand-alone test [7,19].

The practical message is that D-dimer may help exclude AAS only in low-risk patients after structured clinical assessment. A patient with abrupt severe tearing pain, pulse deficit, neurologic deficit, hypotension, known aneurysm, connective tissue disease, aortic valve disease, recent aortic manipulation, malperfusion, syncope, or shock should proceed directly to definitive imaging regardless of D-dimer [19,20]. In a low-risk patient with atypical abdominal or back pain and no high-risk features, D-dimer below 500 ng/mL may reduce the need for CTA when combined with an ADD-RS of 0 or 1 [7,19,20].

## 7. Ruptured Abdominal Aortic Aneurysm

RAAA is a time-critical cause of acute abdomen, back pain, flank pain, collapse, or shock. The diagnostic role of D-dimer in RAAA is limited, because the clinical priority is immediate CTA, if hemodynamically permissible, and vascular surgical management [19,21]. D-dimer may be elevated in aneurysm rupture or contained leak, because of thrombus activation, hemorrhage, and systemic coagulation activation, but a normal D-dimer should never be used to exclude rupture in a patient with compatible symptoms or known aneurysm [21]. In clinical practice, D-dimer has no meaningful rule-out role for suspected RAAA.

## 8. Small Bowel Obstruction, Strangulation, and Intestinal Necrosis

Small bowel obstruction is a common cause of acute surgical abdomen. The major emergency diagnostic challenge is distinguishing simple obstruction from closed-loop obstruction, strangulation, ischemia, necrosis, or perforation [22]. Guidelines emphasize CT as the key imaging modality for identifying the transition point, closed-loop configuration, bowel wall hypo-enhancement, mesenteric edema, pneumatosis intestinalis, portal venous gas, free fluid, and other signs of ischemia [22].

D-dimer has been investigated, because strangulated obstruction causes venous congestion, impaired arterial inflow, endothelial injury, microvascular thrombosis, and bowel wall necrosis [23,24,25]. Bogusevicius et al. prospectively studied 53 patients with small bowel obstruction, and found that D-dimer had a sensitivity of 60%, a specificity of 68%, a positive predictive value of 43%, and a negative predictive value of 81% for strangulated obstruction [23]. In that study, D-dimer was elevated in 60% of strangulated obstructions and 32% of simple obstructions; among strangulated cases, it was abnormal in 71% with bowel necrosis and 43% with reversible ischemia [23]. These values indicate that D-dimer alone is insufficient to diagnose or exclude strangulation.

Yang et al. retrospectively evaluated patients undergoing surgery for acute intestinal obstruction, and reported that combining D-dimer with peritoneal irritation signs improved prediction of intestinal necrosis [24]. More recently, Zhou et al. studied 105 patients with bowel obstruction and found that D-dimer, platelet-to-lymphocyte ratio, and CT signs were associated with intestinal ischemia; the combined model performed better than individual markers [25]. This supports the concept that D-dimer may contribute to multimodal risk stratification, especially when combined with clinical signs and imaging.

In emergency practice, D-dimer may be useful when bowel obstruction is present, and the clinician is uncertain whether ischemia or necrosis is developing. A high D-dimer should increase concern for strangulation, especially when accompanied by continuous severe pain, tachycardia, fever, leukocytosis, metabolic acidosis, elevated lactate, and peritoneal signs [22,24,25]. However, D-dimer should not delay surgery or imaging, and a normal value should not overrule concerning clinical features.

## 9. Incarcerated and Strangulated Hernia

Incarcerated or strangulated hernia is another common cause of acute abdomen and bowel obstruction. Direct clinical evidence for D-dimer in strangulated hernia is limited, compared to small bowel obstruction. The biological rationale is similar: venous congestion, impaired arterial inflow, bowel ischemia, and necrosis may elevate D-dimer [22,23,24,25]. However, diagnosis is primarily clinical and radiological, based on irreducibility, local tenderness, obstructive symptoms, systemic toxicity, and CT findings when needed. D-dimer should not be used to delay urgent surgical assessment in suspected strangulated hernia. If elevated, it may support concern for ischemia, but it does not distinguish strangulated hernia from other causes of acute abdomen.

## 10. Acute Appendicitis

Acute appendicitis remains one of the most common causes of acute surgical abdomen. Diagnosis is based on clinical presentation, inflammatory markers, ultrasonography or CT [1,26]. The 2020 World Society of Emergency Surgery guidelines emphasize structured clinical assessment and appropriate imaging, but no single laboratory test is sufficiently accurate to diagnose appendicitis alone [26].

D-dimer has been evaluated in appendicitis because local inflammation, venous congestion, microthrombosis, gangrene, perforation, abscess, or peritonitis may activate coagulation [10]. Kaya et al. compared D-dimer, procalcitonin, and C-reactive protein in acute appendicitis and found that D-dimer was elevated in only 22 of 78 patients, corresponding to 28.2% [10]. Using a cut-off value of 600 ng/mL FEU, D-dimer was not helpful for differentiating phlegmonous appendicitis from negative appendectomy, whereas C-reactive protein performed better in that comparison [10]. Cayrol et al. studied children and concluded that D-dimer had limited diagnostic accuracy for acute appendicitis, although it might have some prognostic utility [11].

Therefore, D-dimer should not be ordered routinely to diagnose appendicitis. A positive result in right lower quadrant pain is non-specific, and may reflect various inflammatory conditions [4,10,11]. A markedly elevated D-dimer in presumed appendicitis should prompt consideration of complications or alternative diagnoses, including pylephlebitis, MVT, perforation, or sepsis, especially when the clinical course is atypical [12].

## 11. Acute Cholecystitis and Biliary Disease

Acute cholecystitis and biliary colic are common causes of right upper quadrant pain. Diagnosis usually relies on clinical findings, liver function tests, inflammatory markers, ultrasonography, and occasionally hepatobiliary scintigraphy or CT [1,27]. Evidence specifically supporting D-dimer for the diagnosis of acute cholecystitis is sparse. D-dimer may be elevated in severe inflammation, empyema, gangrenous cholecystitis, sepsis, malignancy, PVT, or concomitant thromboembolic disease, but it is not a standard diagnostic marker for biliary disease [4,5]. Consequently, D-dimer should not be used to differentiate biliary colic from acute cholecystitis or to decide on cholecystectomy. Its main value in right upper quadrant pain is indirect: an unexpectedly high value in a patient with atypical symptoms may broaden the differential diagnosis to PE, PVT, malignancy, severe infection, or aortic pathology.

## 12. Acute Pancreatitis

Acute pancreatitis is diagnosed when at least two of three criteria are present: typical abdominal pain, serum amylase or lipase elevation, and characteristic imaging findings [2]. D-dimer is not required for diagnosis. Its value in pancreatitis is mainly prognostic, because severe acute pancreatitis is associated with systemic inflammation, endothelial dysfunction, microcirculatory impairment, organ failure, and coagulation activation [28,29].

Several studies have associated higher D-dimer levels with more severe pancreatitis. Newton et al. reported that a D-dimer level above 933.5 ng/L was suggestive of moderately severe or severe acute pancreatitis with complications [28]. Other studies have reported higher proposed cut-offs, including values around 1871 ng/L for predicting complications, and higher ranges for severe disease, although thresholds vary substantially between cohorts and assays [29]. These data suggest that D-dimer may help identify patients at increased risk of complications, but it should be used alongside established severity assessment, organ failure evaluation, inflammatory markers, imaging, and clinical monitoring [2,28,29].

In the differential diagnosis of acute abdomen, D-dimer elevation in epigastric pain should not be assumed to indicate pancreatitis. It may also occur in AAS, AMI, PE, or bowel ischemia [4,6,20]. Conversely, a normal D-dimer does not exclude pancreatitis. Therefore, D-dimer has no primary diagnostic role in acute pancreatitis, but it may have adjunctive prognostic value once pancreatitis has been diagnosed.

## 13. Acute Diverticulitis

Acute diverticulitis is a common cause of left lower quadrant pain, fever, and inflammatory response. Diagnosis is usually confirmed by CT, particularly when complicated disease, abscess or perforation are suspected [1,30]. Direct evidence supporting D-dimer as a diagnostic marker for diverticulitis is limited. D-dimer may rise in complicated diverticulitis because of inflammation, abscess, perforation, sepsis, or portal pylephlebitis, but these elevations are non-specific [4,5]. Therefore, D-dimer should not be used to diagnose diverticulitis or distinguish uncomplicated from complicated disease. Its value is mainly as a warning biomarker, when markedly elevated values are discordant with presumed uncomplicated diverticulitis. In such cases, clinicians should consider complications, venous thrombosis or malignancy, with CT remaining the key diagnostic test.

## 14. Perforated Hollow Viscus and Peritonitis

Perforated peptic ulcer, perforated diverticulitis, ischemic perforation, and other causes of secondary peritonitis can produce intense systemic inflammation, sepsis, endothelial activation, and coagulation disturbance [1,4,31]. D-dimer may therefore be elevated, especially in severe peritonitis or sepsis. However, there is insufficient evidence to support D-dimer as a primary diagnostic marker for perforation. Diagnosis depends on clinical peritonitis, upright chest or abdominal radiography in selected cases, and CT showing free air, extraluminal contrast, focal bowel wall defect, abscess, or inflammatory source [1]. A high D-dimer in peritonitis should be interpreted as a marker of systemic severity, rather than as proof of perforation. A normal D-dimer should not delay imaging or surgery if perforation is suspected. In this context, lactate, base deficit, organ dysfunction, inflammatory markers, and imaging are more clinically relevant than D-dimer.

## 15. Gynecologic Causes: Adnexal Torsion and Ectopic Pregnancy

Adnexal torsion is a time-sensitive cause of acute lower abdominal or pelvic pain. It involves twisting of the ovary or adnexa around its vascular pedicle, causing venous congestion, impaired lymphatic drainage, arterial compromise, ischemia, and possible necrosis [32,33]. Because venous congestion and ischemia can activate fibrinolysis, D-dimer has been investigated as an adjunctive marker.

Kart et al. demonstrated in an experimental rat model that ovarian torsion produced an acute increase in plasma D-dimer levels, supporting biological plausibility [34]. Topçu et al. evaluated D-dimer in pregnant women with adnexal torsion, and suggested possible diagnostic value, but the study population was very specific and small [35]. More recent pediatric and adolescent data suggest that elevated D-dimer may correlate with adnexal torsion, but the evidence remains preliminary and not sufficient to establish D-dimer as a rule-out test [36].

In emergency practice, D-dimer should not be used to exclude adnexal torsion. Doppler ultrasonography, pregnancy testing, gynecologic assessment, and diagnostic laparoscopy, when suspicion persists, remain central [32,36]. A positive D-dimer may support the possibility of torsion or ischemia, but it is non-specific, particularly in pregnancy [4,5,35]. Regarding ectopic pregnancy, D-dimer has no established diagnostic role; pregnancy testing and transvaginal ultrasonography are decisive.

## 16. Urologic Causes: Renal Colic and Renal Infarction

Renal colic is a common mimic of acute abdomen. D-dimer is not useful for diagnosing ureteric stones. It may be elevated non-specifically in infection, inflammation, malignancy, or concomitant thromboembolic disease [4,5,37]. In uncomplicated renal colic, D-dimer testing is more likely to generate false-positive results than diagnostic clarity.

Renal infarction, however, is a vascular cause of abdominal, flank, or back pain that may be misdiagnosed as renal colic or pyelonephritis [38]. D-dimer may be elevated, because renal infarction is usually embolic or thrombotic, but diagnosis requires contrast-enhanced CT or CTA. In a patient with flank pain, elevated lactate dehydrogenase, AF, embolic risk, hematuria, or unexplained renal dysfunction, an elevated D-dimer may support vascular imaging, but it is not diagnostic [38].

## 17. Pulmonary Embolism and Myocardial Infarction as Extra-Abdominal Mimics

PE may present with upper abdominal pain, flank pain, syncope, nausea, or non-specific discomfort, especially when diaphragmatic pleura is involved or right ventricular strain causes hepatic congestion [4]. In this setting, D-dimer should be used according to validated PE diagnostic pathways, not as a generic acute abdomen test [4,5]. A negative D-dimer can help exclude PE only when pre-test probability is low or intermediate. A positive D-dimer is non-specific and should be interpreted according to probability.

MI may also present with epigastric pain, nausea, vomiting, or abdominal discomfort [39]. D-dimer is not a diagnostic marker for MI. Electrocardiography and cardiac troponin testing are required when cardiac ischemia is possible. Elevated D-dimer in this setting may reflect comorbidity, heart failure, or thrombotic burden, but it should not distract from standard cardiac evaluation [39].

## 18. Proposed Diagnostic Framework and Practical Emergency Department Recommendations

D-dimer should not be used routinely in patients presenting with acute abdominal pain. Instead, its use should be guided by pre-test probability, and a clearly defined diagnostic question. The test is most valuable when a vascular, thrombotic, or ischemic abdominal emergency is suspected, and a negative result could safely reduce the likelihood of disease [3,4,5,7,19,20].

The first step in the emergency department is to identify patients requiring immediate imaging or intervention. Red flags, such as hemodynamic instability, peritonitis, severe pain out of proportion to examination, metabolic acidosis, elevated lactate, AF, known aortic aneurysm, or other features suggestive of AMI or AAS warrant urgent CTA, without delaying management for D-dimer testing [3,18,19,20].

In clinically stable patients, D-dimer may be incorporated into the diagnostic work-up, when AMI, AAS, MVT, PE presenting with abdominal pain, or bowel ischemia remain in the differential diagnosis. For suspected AMI, a negative D-dimer may help exclude disease in carefully selected low-risk patients, whereas patients with moderate or high clinical suspicion should proceed directly to CTA regardless of the result. Similarly, in suspected AAS, D-dimer should only be interpreted together with validated pre-test probability tools, such as the ADD-RS, and should never delay definitive imaging in high-risk patients [3,7,8,18,20].

In bowel obstruction, D-dimer may contribute to identifying patients at increased risk of strangulation or intestinal necrosis, but decisions regarding surgery should continue to rely on clinical assessment, laboratory findings, and CT evidence of ischemia. Current evidence does not support routine D-dimer testing for appendicitis, cholecystitis, diverticulitis, pancreatitis, gastrointestinal perforation, renal colic, or most gynecological emergencies, where it lacks sufficient diagnostic accuracy, even though D-dimer may correlate with disease severity in some of these conditions [22,23,24,25].

**Figure 1:**
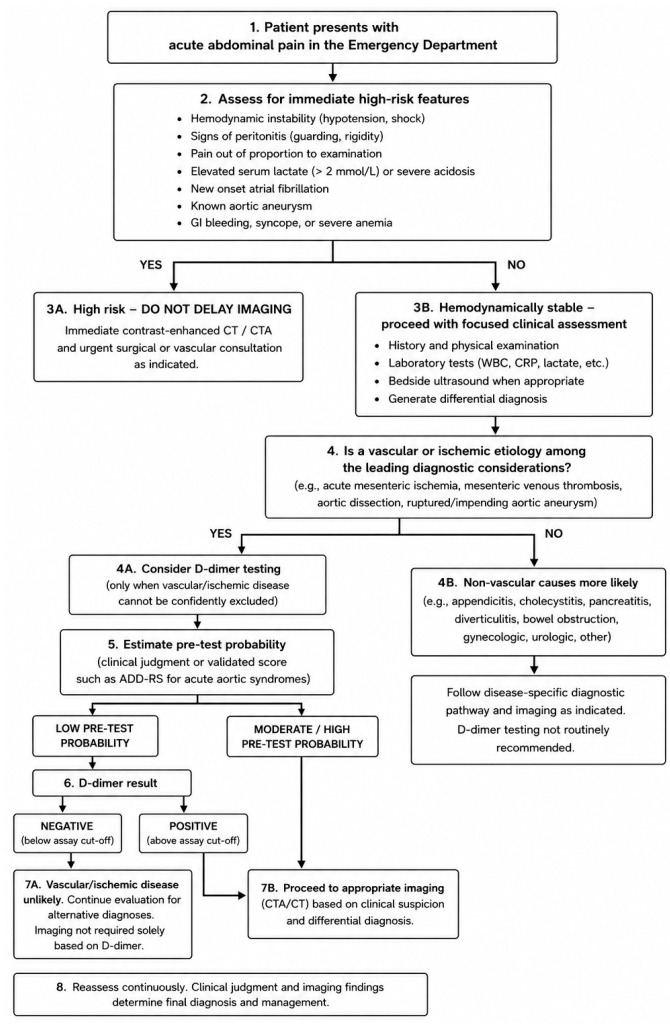
Proposed diagnostic algorithm for D-Dimer testing in patients presenting with acute abdominal pain in the Emergency Department (abbreviations: ADD-RS: Aortic Dissection Detection Risk Score, CRP: C-reactive protein, CT: computed tomography, CTA: computed tomography angiography, GI: gastro-intestinal, WBC: white blood cell count).

Finally, D-dimer results should always be interpreted within the clinical context. A negative result is most informative in patients with low pre-test probability, whereas positive results are non-specific, and frequently occur in elderly patients and those with infection, malignancy, recent surgery, trauma, pregnancy, or systemic inflammation. Overall, D-dimer should be viewed as a selective adjunct to clinical assessment, rather than a screening test, with its greatest value in supporting the evaluation of suspected vascular emergencies, while definitive imaging remains the diagnostic gold standard. This approach preserves the strength of D-dimer as a sensitive adjunct, while avoiding the common error of using it as a non-specific screening test [4,5]. The proposed diagnostic algorithm is summarized in Figure 1.

## 19. Discussion

This review demonstrates that the diagnostic value of D-dimer in patients presenting with acute abdominal pain is largely determined by the underlying pathophysiology, rather than the anatomical diagnosis. Conditions characterized by primary thrombosis or tissue ischemia, including AMI, MVT, and AAS, consistently show the highest diagnostic sensitivity, because activation of coagulation and fibrinolysis is an integral component of disease development [4,6,7,8,17,19,20]. In these settings, D-dimer is best regarded as a rule-out biomarker, that may safely reduce the probability of disease in carefully selected low-risk patients, when interpreted alongside clinical assessment and validated pre-test probability tools [4,6,7,8,17,19,20]. Conversely, patients with moderate or high clinical suspicion should proceed directly to definitive imaging, as recommended by current guidelines [3,18].

The principal limitation of D-dimer is its lack of specificity. Elevated concentrations occur not only in thrombotic disorders, but also in infection, systemic inflammation, malignancy, trauma, pregnancy, recent surgery, advanced age, and critical illness [4,5]. Consequently, many inflammatory causes of acute abdomen, including appendicitis, pancreatitis, diverticulitis, and peritonitis, may produce positive results through secondary activation of coagulation, rather than primary vascular occlusion [10,11,12,28,29]. This explains why D-dimer has limited diagnostic utility in these conditions despite frequent elevation, and reinforces that positive results should never be interpreted in isolation.

The findings of this review also highlight the importance of integrating D-dimer into structured diagnostic pathways, rather than using it as a non-specific screening test. Similar to its established role in VTE, D-dimer is most effective when combined with pre-test probability assessment and appropriate imaging. Its indiscriminate use in unselected patients with abdominal pain is likely to increase false-positive results, unnecessary imaging, and healthcare costs, without improving diagnostic accuracy [4,5]. The proposed diagnostic framework presented in this review therefore emphasizes selective testing based on clinical suspicion, with CT remaining the definitive diagnostic modality, whenever life-threatening vascular pathology cannot be confidently excluded.

Finally, D-dimer should be viewed as one component of a multimodal diagnostic strategy, rather than as a stand-alone biomarker. Emerging evidence suggests that combining D-dimer with serum lactate, inflammatory biomarkers, validated clinical prediction models, and imaging findings may improve diagnostic performance compared with individual tests alone, particularly in patients with suspected vascular abdominal emergencies [15,25]. Future research should therefore focus on validating prospective diagnostic algorithms that combine biomarkers with clinical prediction models and imaging, rather than evaluating isolated biomarker performance alone. The diagnostic value of D-Dimer testing in patients presenting with acute abdominal pain is summarized in Table 1.

## 20. Evidence Limitations and Future Perspectives

The evidence base is limited by heterogeneity in assays, units, cut-offs, reference standards, and timing of sampling. Many studies are retrospective, single-center, and underpowered for uncommon diagnoses. The evidence base is limited by heterogeneity in assays, reporting units (FEU versus DDU), cut-offs, reference standards, and timing of sampling. Spectrum bias is substantial, because D-dimer performs differently in young low-risk outpatients than in elderly hospitalized or septic patients. Publication bias is also possible, especially for smaller biomarker studies. Finally, most studies evaluated diagnostic accuracy, rather than patient-centered outcomes, such as missed ischemia, time to surgery, imaging burden, mortality, or cost-effectiveness.

Future research should focus on prospective emergency department cohorts, standardized D-dimer assays and reporting, disease-specific pre-test probability models, age-adjusted or probability-adjusted thresholds, and integration with lactate, inflammatory markers, imaging findings, and artificial intelligence-supported risk stratification. The most useful studies should test complete diagnostic pathways, rather than isolated biomarker performance.

Finally, another important unresolved issue is the applicability of age-adjusted D-dimer thresholds in acute abdominal emergencies. Although age-adjusted cut-offs are well established for the exclusion of VTE, they have not been validated for acute abdominal pain. Patients with vascular emergencies are typically elderly and often have chronically elevated baseline D-dimer concentrations due to age-related endothelial dysfunction, atherosclerosis, and comorbidities, while inflammatory abdominal disorders independently increase D-dimer through secondary activation of coagulation. These overlapping mechanisms may reduce the diagnostic discrimination achieved by age-adjusted thresholds. Future prospective studies should determine whether disease-specific age-adjusted cut-offs can improve specificity, without compromising the high sensitivity required to exclude life-threatening vascular abdominal emergencies.

## 21. Conclusions

D-dimer is not a general screening test for acute abdominal pain, but a selective adjunct that is most useful when vascular or ischemic abdominal emergencies are suspected. Current evidence supports its greatest diagnostic value in AMI and AAS, where a negative result may contribute to rule-out strategies in carefully selected low-risk patients. In contrast, its role in more common inflammatory, obstructive, and gynecological conditions remains limited by poor specificity and insufficient diagnostic accuracy. D-dimer should therefore always be interpreted in conjunction with clinical assessment, pre-test probability, and appropriate imaging, which remains the cornerstone of diagnosis in patients with suspected life-threatening abdominal pathology.

## Figures and Tables

**Table 1:** Diagnostic value and clinical role of D-dimer across major causes of acute abdominal pain.

Condition	Biological Rationale of D-Dimer Elevation	Diagnostic Performance	Clinical Role
Acute mesenteric ischemia	Arterial or venous thrombosis, bowel ischemia, endothelial injury, tissue necrosis	High sensitivity (94–96%); low specificity (40–50%)	May support rule-out in carefully selected low-risk patients; CTA remains mandatory when clinical suspicion is moderate or high.
Acute aortic syndromes	False lumen thrombosis, activation of coagulation and fibrinolysis	High sensitivity (~95%); intermediate specificity (~60%)	Useful only within validated rule-out strategies combined with pre-test probability
Mesenteric venous thrombosis/Portal vein thrombosis	Venous thrombosis, secondary bowel ischemia	High sensitivity	Supports clinical suspicion, but cannot establish the diagnosis; contrast-enhanced CT is required.
Undifferentiated acute abdomen	Variable ischemia, necrosis, inflammation, coagulation activation	Intermediate diagnostic value	May identify patients with severe intra-abdominal pathology, but does not identify the underlying disease.
Strangulated bowel obstruction/intestinal necrosis	Venous congestion, ischemia, microvascular thrombosis, bowel necrosis	Intermediate sensitivity and specificity	May contribute to risk stratification, when combined with clinical findings and CT.
Acute appendicitis	Local inflammation, microthrombosis, gangrene or perforation	Low diagnostic value	Routine testing is not recommended.
Acute pancreatitis	Systemic inflammation, endothelial dysfunction, coagulation activation	Not established	May correlate with disease severity, but has no primary diagnostic role.
Acute cholecystitis	Severe inflammation, secondary coagulation activation	Low diagnostic value	Routine testing is not recommended.
Acute diverticulitis	Inflammation, abscess, perforation, sepsis	Low diagnostic value	Routine testing is not recommended.
Perforated viscus/secondary peritonitis	Systemic inflammation, endothelial activation, sepsis-associated coagulopathy	Low diagnostic value	Reflects disease severity, rather than diagnosis.
Adnexal torsion	Venous congestion, ischemia, tissue necrosis	Insufficient evidence	Experimental role only; should not influence clinical decision-making.
Renal infarction	Arterial thromboembolism, renal ischemia	Insufficient evidence	May increase suspicion, but diagnosis requires CTA.

Abbreviations: CT: computed tomography, CTA: computed tomography angiography.

## Data Availability

Data is available from the corresponding author.
